# Socio-economic inequalities in health care utilisation in Norway: a population based cross-sectional survey

**DOI:** 10.1186/1472-6963-12-336

**Published:** 2012-09-25

**Authors:** Anne Helen Hansen, Peder A Halvorsen, Unni Ringberg, Olav Helge Førde

**Affiliations:** 1 National Centre for Integrated Care and Telemedicine, University Hospital of Northern Norway, PO box 35, 9038, Tromsø, Norway; 2 National Centre of Rural Medicine and General Practice Research Unit, Department of Community Medicine, University of Tromsø, Tromsø, Norway; 3 Faculty of Health Sciences, Department of Community Medicine, University of Tromsø, Tromsø, Norway

**Keywords:** Cross-sectional study, Socio-economic inequalities, Health care utilisation, General practitioner, Somatic specialist, Psychiatric specialist, Norway

## Abstract

**Background:**

Norway provides universal health care coverage to all residents, but socio-economic inequalities in health are among the largest in Europe. Evidence on inequalities in health care utilisation is sparse, and the aim of this population based study was to investigate socio-economic inequalities in the utilisation of health care services in Tromsø, Norway.

**Methods:**

We used questionnaire data from the cross-sectional Tromsø Study, conducted in 2007–8. All together 12,982 persons aged 30–87 years participated with the response rate of 65.7%. This is slightly more than one third of the total population (33.8%) in the mentioned age group in Tromsø municipality. By logistic regression analyses we studied associations between household income, education and self-rated occupational status and the utilisation of general practitioner, somatic and psychiatric specialist outpatient services. The outcome variables were probability and frequency of use during the previous 12 months. Analyses were stratified by gender and adjusted for age, marital status, and self-rated health.

**Results:**

Self-rated health was the dominant predictor of health care utilisation. Women’s probability of visiting a general practitioner did not vary by socio-economic status, but high income was associated with less frequent use (odds ratio [OR] for trend 0.89, 95% confidence interval [CI] 0.81-0.98). In men, high income predicted lower probability and frequency of general practitioner utilisation (OR for trend 0.85, CI 0.76-0.94, and 0.86, 0.78-0.95, respectively). Women’s probability of visiting a somatic specialist increased with higher income (OR for trend 1.11, CI 1.01-1.21) and higher education (OR for trend 1.27, CI 1.16-1.39). We found the same trends for men, though significant only for education (OR for trend 1.14, CI 1.05-1.25). The likelihood of visiting psychiatric specialist services increased with higher education and decreased with higher income in women (OR for trend 1.57, CI 1.24-1.98, and 0.69, 0.56-0.86, respectively), but did not vary significantly by socio-economic variables in men. Higher income predicted more frequent use of psychiatric specialist services in men (OR for trend 2.02, CI 1.12-3.63).

**Conclusions:**

This study revealed important inequalities in the utilisation of health care services in Norway, inequalities which may contribute to sustaining inequalities in health outcomes.

## Background

Norway is considered one of the best countries to live in with high scores on health parameters [1]. However, national averages conceal inequalities in health outcomes between socio-economic groups, as it is well known that affluent groups have better somatic and mental health and lower mortality than the disadvantaged [2]. Relative health differences between highest and lowest socio-economic groups in Norway are even shown to be among the highest in Europe [3,4], and they seem to be increasing [5]. Although the absolute differences are smaller [2], the Norwegian government has pointed at social inequalities in health and utilisation of health care services as a priority for research and political attention [5].

Health care services are considered one of the explanations for inequalities in health [6,7]. International research from most high-income countries shows a consistent pattern that general practitioner (GP) care is equally or pro-poor distributed while specialist outpatient care tend to favour the better-off [8]. This phenomenon seems stronger where private insurance is common and private specialists make up a significant proportion of available health care [9]. In low- and middle-income countries, utilisation of GP and specialist outpatient care in general tends to be lower among the worse-off, and inequalities larger [10,11].

The Norwegian health care system is based on universal insurance, but an increasing part of the population (about 3.3% or 160000 inhabitants in 2008) has additional private insurance [12]. Primary health care is run by the municipalities. First line medical services are provided to all residents by a regular GP according to the patient list system. Specialist outpatient care is operated by regional and local health enterprises owned by the national government, consisting of public and private somatic and psychiatric specialist services. In this paper the collective term psychiatric specialist services will include visits to psychologists. It also includes visits to hospital staff like nurses and social workers, supervised by psychiatrists and psychologists. Access to specialist care is usually achieved by referral from the GP who acts as a gatekeeper to the specialist health care system. GP and specialist outpatient visits for adults are co-paid by a small fee.

Tromsø is the largest municipality in Northern Norway, with 65,286 inhabitants in January 2008, of whom 38,440 were aged 30–87 years. The municipality is roughly equivalent to Norway as a whole for key parameters like unemployment, income per capita, proportion of disability pensioners, number of primary care physicians per 10,000 residents, and proportion of people living in urban areas, but the level of education in Tromsø is higher than the national average [13]. Tromsø hosts the University Hospital of Northern Norway with inpatient and outpatient clinics. The access to health care services is considered good and roughly in line with Norway as a whole.

Since socio-economic inequalities in health are large in Norway, and evidence on inequalities in health care utilisation is sparse [14], studies in this field are of particular interest. We aimed to examine the socio-economic inequalities in the utilisation of health care services in a more detailed way than previous studies did. Specifically, we tested the hypotheses that initial use and frequent visits to GP, somatic and psychiatric specialist services were associated with socio-economic status in men and women aged 30–87 years in Tromsø, Norway.

## Methods

Population based health surveys have been conducted in Tromsø since 1974. The cross-sectional sixth Tromsø Study (Tromsø 6) was conducted from October 2007 to December 2008, and consisted of comprehensive questionnaires, clinical examination and laboratory tests. The sampling reflected the need for repeated measurements and follow up as well as the need to enrol new participants for ongoing and new projects. As a consequence, four groups were invited; every resident aged 40–42 or 60–87 years (n=12578), a 10% random sample of individuals aged 30–39 (n=1056), a 40% random sample of people aged 43–59 (n= 5787) and all subjects who had attended the second visit of the fourth Tromsø Study, if not already included in the other three groups (n=341). In total 12,982 of 19,762 invited persons aged 30–87 years participated, constituting a response rate of 65.7%. This comprises slightly more than one third of the total population (33.8%) in the mentioned age group in Tromsø municipality.

Our data were collected from the two self-administered questionnaires. The first was mailed with the invitation about two weeks ahead of the suggested appointment time. Those who attended were given the second questionnaire, and most participants completed it while waiting for the clinical examination. Both questionnaires and further details about enrolment methods, attendees and non-attendees are available at the Tromsø Study website [15] and elsewhere [16].

For GP, somatic specialist, and psychiatric specialist services participants were asked if they had visited during the previous year, and if so, how many times. For those who reported to have visited but not the number of visits, we substituted missing values with the average number of visits (given at least one) within each gender and age group. The number of GP visits were stipulated for 627 women (9.0%) and 321 men (5.3%), somatic outpatient visits for 500 women (7.2%) and 308 men (5.1%), and visits to psychiatric specialist services for 81 women (1.2%) and 53 men (0.9%).

For each of the health services, two dichotomous outcome variables were generated, one for no use or use, and one for less frequent or more frequent use. Those who reported at least one visit were grouped as users. Among the users, those with number of visits above the 50th percentile were counted as more frequent users. The more frequent users had 3 or more visits to GP, 2 or more to somatic specialist and 5 or more to psychiatric specialist services in the year prior to the study conduct.

Demographic independent variables were age in 10 year groups and marital status, categorised into married/cohabitant or single living. The following main independent variables were designated as indicators of SES; household’s total gross income in the year prior to the study conduct, education, and self-rated occupational status. Eight income response categories were merged into low income (<200000 NOK), low middle income (201000–400000 NOK), high middle income (401000–700000 NOK) and high income (>700000 NOK). We made three education response categories out of the original five; low (primary and part of secondary school), middle (high school) and high education (college or university). Status of own occupation was rated by the participants from the following sentence: “I consider my occupation to have the following social status in the society”. We merged the five response options (very low - fairly low - middle - fairly high - very high) into three (low - middle - high). Unemployed participants and pensioners were asked to answer for their latest occupation. Response options for the self-rated health variable were reduced from five original categories (very bad - bad - fair - good - excellent) into four by merging the bad and very bad categories due to low numbers in the groups. We validated the self-rated health measure against the five dimensions score scale developed by the Euro Quality of Life Group (EQ-5D) [17], and against dichotomous variables like musculoskeletal pain (for at least three of the previous 12 months), cardiovascular diseases (present or former angina pectoris, heart attack, cerebral stroke/brain haemorrhage, atrial fibrillation and/or high blood pressure), and chronic diseases (present or former cardiovascular disease, asthma, chronic obstructive pulmonary disease, diabetes, mental health problems and/or chronic pain).

Unadjusted visit rates and mean conditional number of visits (given at least one) were obtained by descriptive statistics. We constructed six multivariate logistic regression models each including one of the dependent variables and all independent variables. From three of the models we obtained odds ratios for trends. From the other three we performed dummy analyses to obtain odds ratios for each category in order to identify any lack of linearity in the trend analyses (Tables 1, 2, 3, 4). Due to well-known gender differences in SES and health care utilisation [18], analyses were stratified by gender.

**Table 1:** Women’s probability of health care services utilisation at least once during the previous 12 months*

	**General practitioner n=5184**		**Somatic specialist services n=4956**		**Psychiatric specialist services n=4513**	
	**OR (95% CI)**	**OR for trend (95% CI)**	**OR (95% CI)**	**OR for trend (95% CI)**	**OR (95% CI)**	**OR for trend (95% CI)**
**Marital status**						
Married/cohabitant (1)	0.91 (0.72-1.14)	0.90 (0.72-1.12)	0.77 (0.66-0.91)	0.77 (0.66-0.90)	0.74 (0.50-1.09)	0.75 (0.52-1.10)
Single (0)	1.00		1.00		1.00	
**Household income**						
Low (1)	0.97 (0.64-1.46)	0.97 (0.85-1.09)	0.66 (0.50-0.88)	1.11 (1.01-1.21)	3.82 (1.93-7.57)	0.69 (0.56-0.86)
Low middle (2)	1.07 (0.81-1.43)		0.88 (0.71-1.08)		2.05 (1.18-3.56)	
High middle (3)	0.98 (0.79-1.21)		0.93 (0.79-1.10)		1.68 (1.09-2.59)	
High (4)	1.00		1.00		1.00	
**Education**						
Low (1)	1.16 (0.90-1.49)	0.93 (0.82-1.05)	0.62 (0.51-0.74)	1.27 (1.16-1.39)	0.44 (0.27-0.71)	1.57 (1.24-1.98)
Middle (2)	1.12 (0.92-1.37)		0.88 (0.76-1.02)		0.52 (0.36-0.75)	
High (3)	1.00		1.00		1.00	
**Self-rated occupational status**						
Low (1)	1.12 (0.80-1.55)	0.95 (0.82-1.09)	0.93 (0.74-1.17)	1.04 (0.94-1.15)	1.17 (0.69-1.99)	0.96 (0.74-1.23)
Middle (2)	1.07 (0.90-1.28)		0.93 (0.81-1.06)		1.04 (0.74-1.47)	
High (3)	1.00		1.00		1.00	
**Self -rated health**						
Bad (1)	11.34 (5.49-23.41)	0.48 (0.42-0.54)	4.62 (3.40-6.28)	0.60 (0.55-0.65)	10.82 (5.83-20.08)	0.49 (0.41-0.59)
Fair (2)	4.31 (3.30-5.62)		2.69 (2.22-3.26)		2.83 (1.63-4.94)	
Good (3)	2.01 (1.67-2.42)		1.55 (1.31-1.83)		1.98 (1.19-3.31)	
Excellent (4)	1.00		1.00		1.00	

OR, odds ratio; CI, confidence interval.

* Age adjusted multivariate logistic regressions including all left column variables.

**Table 2:** Women’s frequency of health care services utilisation during the previous 12 months*

	**General practitioner**		**Somatic specialist services**		**Psychiatric specialist services**	
	**OR (95% CI) n=4417**	**OR for trend (95% CI) n=4417**	**OR (95% CI) n=2211**	**OR for trend (95% CI) n=2211**	OR (95% CI) n=195**	OR for trend (95% CI) n=197**
**Marital status**						
Marrried/cohabitant (1)	0.88 (0.74-1.05)	0.85 (0.72-1.01)	0.83 (0.65-1.05)	0.88 (0.70-1.11)	0.59 (0.23-1.49)	0.39 (0.17-0.88)
Single (0)	1.00		1.00		1.00	
**Household income**						
Low (1)	1.39 (1.03-1.89)	0.89 (0.81-0.98)	1.19 (0.77-1.84)	0.96 (0.84-1.10)	0.39 (0.08-1.84)	1.38 (0.89-2.13)
Low middle (2)	1.47 (1.17-1.85)		0.92 (0.67-1.26)		0.56 (0.15-2.04)	
High middle (3)	1.21 (1.01-1.44)		1.07 (0.84-1.37)		0.47 (0.16-1.34)	
High (4)	1.00		1.00		1.00	
**Education**						
Low (1)	1.10 (0.90-1.33)	0.96 (0.87-1.06)	0.95 (0.72-1.24)	1.01 (0.88-1.15)	0.49 (0.15-1.56)	1.43 (0.86-2.37)
Middle (2)	0.97 (0.83-1.14)		1.00 (0.80-1.25)		0.75 (0.30-1.87)	
High (3)	1.00		1.00		1.00	
**Self-rated occupational status**						
Low (1)	0.97 (0.76-1.24)	1.02 (0.91-1.14)	1.06 (0.76-1.48)	0.98 (0.84-1.14)	1.69 (0.44-6.51)	1.00 (0.58-1.73)
Middle (2)	0.95 (0.82-1.10)		1.03 (0.84-1.25)		0.72 (0.31-1.65)	
High (3)	1.00		1.00		1.00	
**Self -rated health**						
Bad (1)	13.82 (9.51-20.10)	0.41 (0.37-0.45)	2.35 (1.55-3.52)	0.74 (0.66-0.83)	4.66 (0.97-22.32)	0.68 (0.46-1.00)
Fair (2)	6.36 (5.08-7.97)		2.11 (1.57-2.83)		1.84 (0.46-7.30)	
Good (3)	2.67 (2.19-3.27)		1.51 (1.15-1.98)		1.48 (0.42-5.28)	
Excellent (4)	1.00		1.00		1.00	

OR, odds ratio; CI, confidence interval.

* Age adjusted multivariate logistic regressions including all left column variables.

**Age group 80–87 years omitted from dummy analyses due to small numbers.

**Table 3:** Men’s probability of health care services utilisation at least once during the previous 12 months*

	**General practitioner n=5123**		**Somatic specialist services n=4959**		**Psychiatric specialist services n=4560**	
	**OR (95% CI)**	**OR for trend (95% CI)**	**OR (95% CI)**	**OR for trend (95% CI)**	**OR (95% CI)**	**OR for trend (95% CI)**
**Marital status**						
Married/cohabitant (1)	1.29 (1.05-1.60)	1.31 (1.07-1.61)	0.98 (0.82-1.17)	0.99 (0.83-1.18)	0.85 (0.53-1.37)	0.82 (0.51-1.30)
Single (0)	1.00		1.00		1.00	
**Household income**						
Low (1)	1.14 (0.76-1.72)	0.85 (0.76-0.94)	0.69 (0.50-0.95)	1.08 (0.99-1.18)	1.55 (0.70-3.43)	0.89 (0.70-1.15)
Low middle (2)	1.40 (1.09-1.80)		0.90 (0.73-1.11)		1.05 (0.58-1.89)	
High middle (3)	1.18 (1.00-1.41)		0.96 (0.82-1.12)		0.83 (0.52-1.33)	
High (4)	1.00		1.00		1.00	
**Education**						
Low (1)	1.00 (0.81-1.23)	1.00 (0.90-1.11)	0.77 (0.65-0.92)	1.14 (1.05-1.25)	0.80 (0.49-1.32)	1.12 (0.88-1.44)
Middle (2)	1.17 (0.99-1.38)		0.92 (0.80-1.06)		0.74 (0.48-1.14)	
High (3)	1.00		1.00		1.00	
**Self-rated occupational status**						
Low (1)	1.06 (0.75-1.49)	1.06 (0.93-1.20)	0.91 (0.69-1.19)	1.11 (0.99-1.23)	1.25 (0.65-2.44)	1.00 (0.74-1.35)
Middle (2)	0.88 (0.76-1.04)		0.86 (0.76-0.98)		0.92 (0.63-1.36)	
High (3)	1.00		1.00		1.00	
**Self -rated health**						
Bad (1)	11.95 (5.77-24.77)	0.52 (0.47-0.58)	5.52 (3.93-7.76)	0.60 (0.55-0.66)	11.72 (4.77-28.77)	0.42 (0.33-0.53)
Fair (2)	3.40 (2.69-4.29)		2.46 (2.00-3.03)		5.37 (2.40-11.99)	
Good (3)	1.49 (1.24-1.78)		1.54 (1.27-1.86)		2.05 (0.92-4.57)	
Excellent (4)	1.00		1.00		1.00	

OR, odds ratio; CI, confidence interval.

* Age adjusted multivariate logistic regressions including all left column variables.

**Table 4:** Men’s frequency of health care services utilisation during the previous 12 months*

	**General practitioner**		**Somatic specialist services**		**Psychiatric specialist services**	
	**OR (95% CI) n=4005**	**OR for trend (95% CI) n=4005**	**OR (95% CI) n=1902**	**OR for trend (95% CI) n=1902**	OR (95% CI) n=136**	OR for trend (95% CI) n=139**
**Marital status**						
Married/cohabitant (1)	0.95 (0.78-1.16)	0.95 (0.78-1.15)	0.79 (0.60-1.05)	0.82 (0.62-1.09)	0.07 (0.02-0.33)	0.08 (0.02-0.31)
Single (0)	1.00		1.00		1.00	
**Household income**						
Low (1)	1.74 (1.24-2.46)	0.86 (0.78-0.95)	0.80 (0.48-1.34)	0.99 (0.86-1.14)	0.37 (0.05-3.08)	2.02 (1.12-3.63)
Low middle (2)	1.33 (1.06-1.67)		1.05 (0.76-1.46)		0.18 (0.04-0.81)	
High middle (3)	1.20 (1.01-1.43)		1.16 (0.91-1.48)		0.45 (0.15-1.34)	
High (4)	1.00		1.00		1.00	
**Education**						
Low (1)	1.28 (1.05-1.55)	0.87 (0.79-0.96)	1.08 (0.82-1.44)	0.96 (0.83-1.10)	2.62 (0.75-9.18)	0.60 (0.33-1.08)
Middle (2)	1.32 (1.13-1.55)		1.11 (0.88-1.40)		1.35 (0.45-4.04)	
High (3)	1.00		1.00		1.00	
**Self-rated occupational status**						
Low (1)	1.01 (0.75-1.36)	1.00 (0.88-1.12)	0.59 (0.39-0.91)	1.18 (1.00-1.40)	0.16 (0.03-1.00)	2.01 (0.96-4.18)
Middle (2)	0.97 (0.84-1.13)		0.90 (0.73-1.11)		0.39 (0.14-1.10)	
High (3)	1.00		1.00		1.00	
**Self -rated health**						
Bad (1)	8.01 (5.46-11.77)	0.48 (0.44-0.53)	3.68 (2.24-6.05)	0.63 (0.56-0.72)	4.82 (0.65-35.90)	0.69 (0.41-1.14)
Fair (2)	4.03 (3.13-5.19)		2.00 (1.42-2.82)		6.62 (0.97-44.92)	
Good (3)	1.84 (1.45-2.34)		1.15 (0.84-1.59)		4.38 (0.65-29.44)	
Excellent (4)	1.00		1.00		1.00	

OR, odds ratio; CI; confidence interval.

* Age adjusted multivariate logistic regressions including all left column variables.

** Age group 80–87 years omitted from dummy analyses due to small numbers.

We used 95% Confidence intervals (CI) throughout the study. All analyses were done in Stata, version 12.0. The Tromsø Study as well as the protocol for this particular project has been approved by The Regional Committee of Research Ethics.

## Results

### Sample characteristics

There were more women in the sample (53.4%), and also more women than men lived in lower income and single person households. The highest percentage of people had higher education, good health, and belonged to high middle income households (Table 5).

**Table 5:** Characteristics of the participants (%) stratified by gender and utilisation of health care services*

	**Women**				**Men**			
	**Total sample n=6929**	**GP n=5897**	**Somatic specialist services n=2857**	**Psychiatric specialist services n=278**	**Total sample n=6053**	**GP n=4727**	**Somatic specialist services n=2194**	**Psychiatric specialist services n=179**
**Age**	n=6929	n=5897	n=2857	n=278	n=6053	n=4727	n=2194	n=179
30-39	4.3	4.3	4.0	7.9	3.5	3.2	2.7	1.7
40–49	27.6	26.4	26.1	39.9	27.5	23.8	20.8	28.5
50–59	18.6	18.1	19.3	16.9	18.9	18.4	18.5	21.2
60–69	30.4	30.7	30.9	20.5	33.0	34.6	37.0	27.4
70–79	14.3	15.3	14.6	11.2	13.9	16.3	17.5	17.9
80-87	4.8	5.2	5.1	3.6	3.2	3.7	3.5	3.3
**Marital status**	n=6627	n=5649	n=2745	n=266	n=5932	n=4645	n=2160	n=170
Married/ cohabitant	68.8	68.2	67.1	59.8	82.1	82.4	82.8	74.7
Single	31.2	31.8	32.9	40.2	17.9	17.6	17.2	25.3
**Household income**	n=6180	n=5248	n=2602	n=245	n=5788	n=4509	n=2118	n=171
Low	16.0	17.0	14.6	22.9	8.1	8.9	7.7	17.5
Low middle	29.0	29.8	30.6	28.6	24.2	26.1	26.5	28.7
High middle	31.9	31.5	32.3	31.0	38.8	38.7	38.7	29.8
High	23.1	21.7	22.5	17.5	28.9	26.3	27.1	24.0
**Education**	n=6824	n=5807	n=2825	n=275	n=5975	n=4669	n=2178	n=175
Low	31.9	33.1	27.8	28.7	25.0	26.3	23.7	29.7
Middle	31.8	32.3	33.4	26.2	35.5	36.3	35.6	32.0
High	36.3	34.6	38.8	45.1	39.5	37.4	40.7	38.3
**Self-rated status of occupation**	n=5967	n=5061	n=2486	n=226	n=5498	n=4280	n=2008	n=156
Low	10.0	10.4	9.7	13.7	5.6	5.9	5.7	8.3
Middle	59.1	59.5	58.5	55.3	52.2	52.5	50.1	50.7
High	30.9	30.1	31.8	31.0	42.2	41.6	44.2	41.0
**Self-rated health**	n=6855	n=5839	n=2834	n=277	n=6009	n=4697	n=2184	n=178
Bad	6.0	6.9	8.4	18.1	4.8	5.8	8.2	14.6
Fair	29.3	31.6	33.9	33.2	28.2	31.6	33.6	43.3
Good	49.4	48.7	45.8	39.7	53.3	51.4	48.9	37.6
Excellent	15.3	12.8	11.9	9.0	13.7	11.2	9.3	4.5

GP, general practitioner.

* One or more visits during the previous 12 months.

The likelihood of a visit to the health care services tended to increase by poorer health and age, but women’s visits to psychiatric specialist tended to decrease by age (Table 6).

**Table 6:** Proportion of participants visiting health care services at least once during the previous 12 months

	**Women**			**Men**		
	**General practitioner n=6844**	**Somatic specialist services n=6401**	**Psychiatric specialist services n=5818**	**General practitioner n=6006**	**Somatic specialist services n=5753**	**Psychiatric specialist services n=5304**
	**Rate/100 (95% CI)**	**Rate/100 (95% CI)**	**Rate/100 (95% CI)**	**Rate/100 (95% CI)**	**Rate/100 (95% CI)**	**Rate/100 (95% CI)**
**Total**	86.2 (85.3-87.0)	44.6 (43.4-45.9)	4.8 (4.2-5.3)	78.7 (77.7-79.7)	38.1 (36.9-39.4)	3.4 (2.9-3.9)
**Age**	n=6844	n=6401	n=5818	n=6006	n=5753	n=5304
30-39	85.5 (81.4-89.5)	40.6 (34.9-46.4)	8.4 (5.0-11.7)	72.4 (66.3-78.4)	28.6 (22.5-34.8)	1.5 (−0.2-3.2)
40–49	82.3 (80.6-84.0)	41.0 (38.7-43.2)	6.5 (5.3-7.7)	68.3 (66.0-70.5)	28.3 (26.1-30.5)	3.3 (2.4-4.2)
50–59	83.5 (81.4-85.5)	44.8 (42.1-47.6)	4.2 (3.0-5.4)	76.6 (74.1-79.0)	36.8 (34.0-39.7)	3.7 (2.5-4.8)
60–69	86.9 (85.5-88.4)	45.5 (43.3-47.8)	3.3 (2.5-4.2)	82.3 (80.7-84.0)	42.7 (40.5-45.0)	2.9 (2.1-3.7)
70–79	93.2 (91.6-94.8)	48.7 (45.3-52.0)	4.1 (2.7-5.5)	92.2 (90.4-94.0)	49.5 (46.0-53.0)	4.7 (3.1-6.3)
80-87	93.9 (91.3-96.5)	53.3 (47.4-59.2)	4.1 (1.6-6.6)	91.7 (87.8-95.6)	48.7 (40.9-56.6)	4.1 (0.9-7.2)
**Marital status**	n=6558	n=6160	n=5620	n=5897	n=5660	n=5218
Married/cohabitant	85.3 (84.3-86.3)	43.1 (41.6-44.6)	4.1 (3.5-4.7)	79.1 (77.9-80.2)	38.4 (37.0-39.8)	3.0 (2.4-3.5)
Single	88.0 (86.6-89.4)	47.8 (45.6-50.1)	6.2 (5.1-7.4)	77.5 (75.0-80.0)	37.1 (34.1-40.1)	4.7 (3.3-6.0)
**Self-rated health**	n=6781	n=6349	n=5776	n=5969	n=5723	n=5275
Bad	97.3 (95.8-98.9)	63.9 (59.0-68.8)	15.4 (11.5-19.3)	96.1 (93.9-98.4)	65.9 (60.3-71.6)	11.1 (7.0-15.1)
Fair	93.3 (92.2-94.4)	53.0 (50.7-55.3)	5.8 (4.6-6.9)	88.0 (86.4-89.5)	46.1 (43.7-48.6)	5.4 (4.2-6.6)
Good	85.0 (83.8-86.2)	41.2 (39.5-42.9)	3.8 (3.1-4.5)	75.9 (74.4-77.4)	35.0 (33.3-36.7)	2.4 (1.8-2.9)
Excellent	71.7 (68.9-74.4)	33.3 (30.4-36.2)	2.6 (1.6-3.6)	64.3 (61.1-67.6)	25.2 (22.2-28.2)	1.0 (0.3-1.8)

CI, confidence interval.

The overall mean conditional number of visits (given at least one) to GP in the year prior to the study conduct was 3.4 for women (Confidence Interval [CI] 3.3-3.5) and 3.1 for men (CI 3.1-3.2), to somatic specialist 2.4 for women (CI 2.3-2.5) and 2.4 for men (CI 2.3-2.6), and to psychiatric specialist services 10.0 for women (CI 8.2-11.6) and 6.6 for men (CI 5.5-7.8).

### Visit to general practitioner

Women’s probability of visiting a GP once or more during the year did not vary by SES (Table 1), but a trend towards less frequent use with higher income was observed (OR for trend 0.89, CI 0.81-0.98) (Table 2).

Compared to the highest income group, women with low middle income had the highest odds of frequent use (OR 1.47, CI 1.17-1.85).

In men, high income was associated with lower probability (OR for trend 0.85, CI 0.76-0.94) (Table 3) and lower frequency of GP visits (OR for trend 0.86, CI 0.78-0.95) (Table 4). The lowest income group deviated from the trend as the probability of visiting was not different from the highest income group (OR 1.14, CI 0.76-1.72).

As for women, men’s likelihood to seek primary care was not associated with self-rated occupational status or education (Tables 1 and 3). Compared to the highest educated men, those with middle education had the highest odds of frequent use (OR 1.32, CI 1.13-1.55) (Table 4). Married men/cohabitants were more likely to make a visit than single men (Table 3), but there was no significant association between marital status and the frequency of visits (Table 4).

### Visit to somatic specialist

Women’s probability of visiting a somatic specialist increased with higher income (OR for trend 1.11, CI 1.01-1.21), and higher education (OR for trend 1.27, CI 1.16-1.39) (Table 1). Married women/cohabitants were less likely to make a visit than singles (Table 1). For men, the probability of visiting a somatic specialist increased significantly with higher education (OR for trend 1.14, CI 1.05-1.25) (Table 3). Men in the lowest income group were less likely to visit compared to men with high income (OR 0.69, CI 0.50-0.95). None of the socio-economic variables predicted more frequent use in either gender, with the exception that men who rated their occupational status as low were less likely to visit somatic specialist services frequently (OR 0.59, CI 0.39-0.91) (Tables 2 and 4).

### Visit to psychiatric specialist services

Women’s probability of visiting psychiatric specialist services increased with higher education (OR for trend 1.57, CI 1.24-1.98) but decreased with higher income (OR for trend 0.69, CI 0.56-0.86) (Table 1), whereas for men we found no significant associations with the socio-economic variables (Table 3). Men’s frequency of visits to psychiatric specialist services increased with higher income (OR for trend 2.02, CI 1.12-3.63) (Table 4). In dummy analyses however, only the low middle income group differed significantly from the high income reference (OR 0.18, CI 0.04-0.81). Single living predicted more frequent visits in both genders, but neither education nor self-rated occupational status had any significant impact on the frequency of visits (Tables 2 and 4).

### Self-rated health

Participants who rated their health as bad had a higher probability and frequency of visits to all health care services, with the exception of men’s frequency of visits to psychiatric specialist services (Tables 1, 2, 3, 4). By substituting self-rated health with variables like musculoskeletal pain, cardiovascular diseases, chronic diseases, or the EQ-5D scale our results were the same (data not shown).

## Discussion

Even if self-rated health was the strongest predictor for use of health care services, we have shown important inequalities in the utilisation according to SES in a country where relative health differences are considered large between SES groups [3,4], and where equitable access to health care services is a political objective [5] and even a statutory right [19].

Five key points stand out. First, GP services utilisation was significantly higher in lower SES groups, where the greatest needs are likely to be found. Smaller sample studies from Norway report similar non-significant trends [8,20,21], or no trends [22]. Our findings were the most consistent for men, and apply both to the initial and subsequent visits. For lower income women this applies to frequency but not to the initial visit, possibly due to regular gynaecological and preventive GP consultations leading to levelling between SES groups.

Second, GP services utilisation seemed highest in the low middle income group, a tendency found in the study by Jensen as well [22]. We made similar findings for education, but only in men. Since health status follows a continuous gradient from the better-off to the worse-off, this break of the curve is not likely to reflect differences in need. In Sweden, more people in the lowest income group reported needing but not seeking care [23]. In fact, looking at the lower income and educational groups separately, the utilisation profile may resemble middle- and low-income countries [10,11], indicating barriers due to absolute rather than relative deficiencies. Financial (user fees, transport costs), organisational (inflexible service delivery) and cultural barriers (language, communication, stigma) might have an impact [6,23]. Difficulty in paying for health care has been reported for 7% of patients in Norway [24]. For men in the lowest income group the probability of an initial visit was lower, not only to a GP, but also to a somatic specialist (Table 3). In a gate-keeper system the initial GP visit is crucial, making up the basis for access to additional care.

Third, the probability of an initial visit to a somatic specialist was higher among the richer and better educated, first and foremost for women. This is noteworthy since health is worse in lower SES groups. Our findings are consistent with previous research [8,20,22] and the inverse care law [25]. Former studies from Norway have shown the same tendency for private but not consistently for public specialist visits [21,26]. Supplementary private insurance could explain some, but not all of this pro high SES bias [12]. Like others, we found no evidence that frequency of somatic specialist visits was influenced by SES [8,20-22].

Fourth, the probability of an initial psychiatric specialist visit was higher for the better educated, although significant only in women. This contrasts with the general suggestion of higher utilisation in deprived groups [27,28], but corresponds to findings from Norway [22] and the US [29]. A possible explanation could be that higher educated groups and women might recognise and accept psychiatric needs more than lower educated groups and men [29]. The opposite trend for income might be a sign of minimal financial barriers, but possibly also an expression of negative influence by psychiatric disease on income (social selection) [30].

Fifth, since lower SES groups were more frequent users of GP services and less likely to make an initial specialist visit, it may be that more additional somatic care tends to take place in primary services for disadvantaged groups and in specialist services for the better-off. This applies particularly to women (Tables 1 and 2). GP referrals might thus be biased according to SES and gender. This interpretation is in accordance with former findings of higher GP referral rates for the better or more educated [22,31], and later referral of women [32]. On the other hand, disadvantaged groups may miss out on attending additional somatic care. Also, affluent groups may bypass the GP and achieve specialist care directly. Our cross-sectional data cannot indicate the reasons for these differences in the utilisation of GP and specialist care, neither infer whether follow-up in primary or specialist care is preferable, and for whom.

Our study had several limitations. Despite a high response rate, our sample may not be entirely representative of the general population, as it is well known that women, married/cohabitants, higher socio-economic groups and healthier persons are more likely to participate in population surveys [33]. In Tromsø 6, attendees were older, and the proportions of married/cohabitants and women were higher than in non-attendees [15,16]. Also, as reflected in our sample the level of education in Tromsø is higher than for Norway as a whole (Table 5) [34], and thus generalisation must be made with caution. Further, the validity of self-reported data may be questioned, but agreement between self-reported and registered utilisation of health care is generally high [35]. One should also bear in mind the potential for recall bias and underreporting, which will probably be largest for psychiatric services utilisation. We were not able to adjust household income for number in household, though it was adjusted for marital status. A cross-sectional study like the present cannot establish causal relationships, nor explore the quality of care. Finally, we cannot exclude the possibility of unmeasured confounders of the associations between SES and health care utilisation.

Particular strengths of this study were the large sample size, which allowed us to study somatic and psychiatric outpatient visits, and gender, separately. Furthermore, the comprehensive coverage of relevant issues in the questionnaires made it possible to validate variables against each other and to some extent compensate for recall bias and underreporting. For instance, validations of self-rated health made us consider it robust and preferable to adjust for self-rated health in all models, even if this is not an indisputable measure of need [36]. Also, education was a robust variable in our sample since most of the participants had completed their education. The geographic location and availability of health services made Tromsø particularly suitable for this study.

## Conclusions

Our findings strongly suggest that the utilisation of general practice is higher in lower SES groups and that the inverse care law still rules regarding the utilisation of somatic and psychiatric outpatient specialist services in Norway. The reasons for socio-economic inequalities in health and health care utilisation, and whether health care services may sustain inequalities in health outcomes, should be explored in further studies.

## Abbreviations

CI: Confidence Interval; EQ-5D: Euro Quality of Life Group five Dimensions score scale; GP: General Practitioner; NOK: Norwegian Kroner; OECD: Organisation for Economic Cooperation and Development; OR: Odds ratio; SES: Socio Economic Status; Tromsø 6: The sixth Tromsø Study; US: United States; WHO: World Health Organization.

## Competing interests

The authors declare that they have no competing interests.

## Authors’ contributions

All the authors contributed to the design and conduct of the study. AHH did the statistical analyses. All authors contributed substantially to the discussion of results. AHH drafted the manuscript. OHF, PAH and UR contributed with major improvements and critical revisions. All the authors approved the final version for publication.

## Pre-publication history

The pre-publication history for this paper can be accessed here:

http://www.biomedcentral.com/1472-6963/12/336/prepub
